# Investigations on the interplays between *Schistosoma mansoni*, praziquantel and the gut microbiome

**DOI:** 10.1186/s13071-018-2739-2

**Published:** 2018-03-12

**Authors:** Pierre H. H. Schneeberger, Jean T. Coulibaly, Gordana Panic, Claudia Daubenberger, Morgan Gueuning, Jürg E. Frey, Jennifer Keiser

**Affiliations:** 1 Department of Medical Parasitology and Infection Biology, Swiss Tropical and Public Health Institute, Basel, Switzerland; 2 University of Basel, Basel, Switzerland; 3 Department of Methods Development and Analytics, Agroscope, Wädenswil, Switzerland; 4 Unité de Formation et de Recherche Biosciences, Université Felix Houphouët-Boigny, Abidjan, Côte d’Ivoire; 5 Centre Suisse de Recherches Scientifiques en Côte d’Ivoire, Abidjan, Côte d’Ivoire

**Keywords:** *Schistosoma mansoni*, Praziquantel, Gut microbiome, Infectious disease, Microbiome-parasite interaction, Microbiome-drug interaction

## Abstract

**Background:**

Schistosomiasis is a neglected tropical disease burdening millions of people. One drug, praziquantel, is currently used for treatment and control. Clinically relevant drug resistance has not yet been described, but there is considerable heterogeneity in treatment outcomes, ranging from cure to only moderate egg reduction rates. The objectives of this study are to investigate potential worm-induced dysbacteriosis of the gut microbiota and to assess whether a specific microbiome profile could influence praziquantel response.

**Methods:**

Using V3 and V4 regions of *16S* rRNA genes, we screened the gut microbiota of 34 *Schistosoma mansoni* infected and uninfected children from Côte d’Ivoire. From each infected child one pre-treatment, one 24-hour and one 21-day follow-up sample after administering 60 mg/kg praziquantel or placebo, were collected.

**Results:**

Overall taxonomic profiling and diversity indicators were found to be close to a “healthy” gut structure in all children. Slight overall compositional changes were observed between *S. mansoni*-infected and non-infected children. Praziquantel treatment was not linked to a major shift in the gut taxonomic profiles, thus reinforcing the good safety profile of the drug by ruling out off-targets effects on the gut microbes.*16S* rRNA gene of the *Fusobacteriales* order was significantly more abundant in cured individuals, both at baseline and 24 hours post-treatment. A real-time qPCR confirmed the over-abundance of *Fusobacterium* spp. in cured children. *Fusobacterium* spp. abundance could also be correlated with treatment induced *S. mansoni* egg-reduction.

**Conclusions:**

Our study suggests that neither a *S. mansoni* infection nor praziquantel administration triggers a significant effect on the microbial composition and that a higher abundance of *Fusobacterium* spp., before treatment, is associated with higher efficacy of praziquantel in the treatment of *S. mansoni* infections.

**Trial registration:**

International Standard Randomised Controlled Trial, number ISRCTN15280205.

**Electronic supplementary material:**

The online version of this article (10.1186/s13071-018-2739-2) contains supplementary material, which is available to authorized users.

## Background

Schistosomiasis belongs to the group of neglected tropical diseases caused by parasitic worms of the genus *Schistosoma* [1]. Schistosomes have accompanied mankind for thousands of years and - still today - they are pervasive where poverty prevails [2, 3]. Recent estimates suggest that approximately 200 million people are affected by schistosomiasis [1]. Left untreated, the disease becomes chronic and debilitating and is therefore known as one of the diseases that perpetuate the “poverty trap” [4, 5]. *Schistosoma mansoni* inhabits the mesenteric veins of the gut and, by releasing eggs, triggers a host cellular immune response, causing a wide range of clinical manifestations including serious gut inflammation [1]. Similarly, to other parasitic worms, they interact and affect the same environment as the gut microbiota [6, 7].

Treatment options against schistosomiasis are limited to only one drug, namely, praziquantel. Developed in the mid-1970s, praziquantel remains poorly characterized. Its mechanism of action is not well understood and pharmacokinetic/dynamic relationships have not yet been determined [8, 9]. Remarkably, there is considerable heterogeneity in treatment outcomes, yet the link to any type of resistance mechanism remains elusive [1, 10, 11]. Hence, it is possible that the efficacy of praziquantel is dependent on a variety of factors, including - but not limited - to hosts genetic background, differences in the drug disposition and bioavailability, and the gut microbiota [6, 7].

Several studies have aimed at characterizing the microbiome from different parts of the human body and have shown considerable variation from one individual to another [12–14]. The gut microbiome is composed of hundreds of different microorganisms, including eukaryotic parasites, bacteria and viruses [15–17]. Bacteria are the most predominant type, with approximately 300 to 1000 different species in the intestine, and account for a large majority of the genetic material present in stool [16, 18, 19]. Recent studies have shown that specific composition of the microbiota, i.e. species diversity as well as relative abundance, modulates the metabolism and disposal of xenobiotics [20–24].

In this study, we investigate the associations between *S. mansoni*, praziquantel and the gut microbial composition in the framework of a randomized, controlled, dose-finding and pharmacokinetic trial of praziquantel in preschool-aged and school-aged children. Samples were analysed to explore differences in microbial composition between (i) infected and non-infected children, (ii) those receiving praziquantel *versus* placebo, and (iii) those for which treatment with praziquantel resulted either in successful or unsuccessful clearance of worms.

## Methods

### Sample collection

The stool samples used in this study were sourced from a praziquantel dose-finding clinical trial in school-aged and pre-school-aged in Côte d’Ivoire [25]. Briefly, the clinical trial was executed between November 2014 and February 2015 in five villages located in the health district of Azaguié, southern Côte d’Ivoire. *Schistosoma mansoni*-positive children (*n* = 317; 2–15 years) as confirmed by quadruplicate Kato-Katz smears from 2 stool samples were eligible to participate in this trial and randomized to 20, 40 or 60 mg/kg praziquantel or placebo. For this study, a sub-set of samples from 34 children treated with 60 mg/kg were selected for microbial analysis. Children had been tested for *Ascaris lumbricoides*, hookworm and *Trichuris trichuria*, and those who were co-infected with either of these parasites were not included in the subset of 34 children analysed in this study. Children who received anthelminthic or antimalarial treatment, or presented symptoms of a systemic disease 4 weeks prior to this study were excluded. From each child, stool samples were collected prior to treatment (labelled with “A”), 24 hours post-treatment (labelled with “B”) and 3 weeks post-treatment (labelled with “C”), and a total of 96 stool samples were analysed. Samples from each of these time-points were selected according to four different conditions (see Table 1): (1) non-infected children; (2) *S. mansoni* infection and cure of children following 60 mg/kg praziquantel administration; (3) *S. mansoni* infection and children not cured following 60 mg/kg praziquantel administration; (4) children infected with *S. mansoni* receiving a placebo. The *S. mansoni-*positive groups (Groups 2–4) were further stratified according to infection intensity as defined by the World Health Organization (WHO) guidelines for classification of schistosomiasis: (i) high (over 400 eggs per gram (epg) of stool); (ii) moderate (100–399 epg); or (iii) low (1–99 epg) [26]. We included children characterized by different infection intensities (Table 1).

**Table 1:** Summary of participants investigated in this study. Each child provided a sample before, 24 hours after and 3 weeks after treatment with single dose (60 mg/kg) dose of praziquantel. For the placebo controls, samples were collected at the same time points

*S. mansoni* infection	Infection intensity (epg)	Treatment outcome	No. of participants	Sample identifiers	Group name
Negative	0	Not applicable	6	1; 12; 13; 14; 15; 25	Controls
Positive	1–99	Cure	0		Cure
	100–399		5	11; 17; 22; 26; 28	
	> 400		4	2; 7; 16; 27	
	1–99	Not cured	3	19; 23; 32	Not cured
	100–399		3	10; 30; 31	
	> 400		2	5; 8	
	1–99	Placebo	3	20; 29; 34	Placebo
	100–399		5	6; 18; 21; 24; 33; 1	
	> 400		3	3; 4; 9	

*Abbreviation*: *epg* eggs per gram stool

The subset of children was selected based on the availability of (i) complete metadata and demographic information, (ii) all parasitology test results, and (iii) the three stool samples, at all three sampling times.

Pre-schoolers (3–5 year-old) had a Body Mass Index (BMI) ranging from 12.7 to 21 and school-aged children (5–13 year-old) had a BMI ranging from 14.7 to 32.2. Detailed information is summarized in Additional file 1: Table S1. According to thresholds set by the WHO, growth indicators indicate that nutritional stunting is not occurring in the subset of children involved in this study and that the potential impact of malnutrition on the gut microbiota can be ruled out.

### DNA isolation

For each sample, 150 mg of faecal material was used to isolate genomic DNA. DNA isolation was conducted with the QIAamp DNA Stool kits (Qiagen, Hilden, Germany) according to the manufacturers’ recommendations. Extracted DNA was eluted in 50 μl of the elution buffer and used for downstream analyses. Concentrations were measured with a Qubit 3.0 fluorometer using a dsDNA High-sensitivity Assay kit (Thermo Fisher Scientific, Waltham, MA, USA). All concentrations are indicated in Additional file 2: Table S2.

### *16S* amplicon polymerase chain reaction (PCR)

Isolated DNA (2.5 μl) was used to perform amplification of the V3-V4 region using the following primer pair, according to Klindworth et al. [27]: forward (5'-TCG TCG GCA GCG TCA GAT GTG TAT AAG AGA CAG CCT ACG GGN GGC WGC AG-3'); reverse (5'-GTC TCG TGG GCT CGG AGA TGT GTA TAA GAG ACA GGA CTA CHV GGG TAT CTA ATC C-3').

The reaction was performed in 25 μl reaction volumes using the 2X KAPA HiFi HotStar ReadyMix (KAPA Biosystems, Boston, MA, USA). The thermocycler was set to the following parameters: 95 °C for 3 min, 25 cycles of 95 °C (30 s), 55 °C (30 s) and 72 °C (30 s), one additional step at 72 °C (5 min) and finally set on hold indefinitely at 4 °C. The quality of the amplified product was controlled visually on a 1% agarose gel. The amplicons were purified with an AMPure XP beads (Beckman-Coulter, Fullerton, CA, USA) protocol.

### Sequencing

The barcoding PCR was performed using primer pairs from the Nextera XT Index kit (Illumina, San Diego, CA, USA) to produce 96 amplicon pools with different tags. The amplification reaction was conducted with the same reagents and the thermocycler was set to the following parameters: 95 °C for 3 min, 20 cycles of 95 °C (30 s), 55 °C (30 s), 72 °C (30 s), one additional step at 72 °C (5 min) and a final step at 4 °C until further processing. Similarly to the first PCR, amplified products were cleaned with AMPure XP beads. The quality of the product was assessed using a 1% agarose gel and the quantification was performed using a Qubit 3.0 Fluorimeter (Thermo Fisher Scientific, Waltham, MA, USA) and the corresponding High-sensitivity dsDNA HS Assay Kit. The 96 amplicon samples were pooled together in an equimolar way and loaded on a cartridge on the Illumina MiSeq sequencing system (Illumina, USA). MiSeq Reagent Kit V3 (2 × 300 bp) sequencing reagents (Illumina, USA) were used for this experiment.

### *Fusobacterium* spp. quantitative real-time PCR (qPCR)

*Fusobacterium* spp. was amplified using the primers and Taqman probes as described in Martin et al. [28]. DNA from *Fusobacterium nucleatum* was obtained from the German Collection of Microorganisms and Cell Cultures (DSMZ). Briefly, 2 μl of isolated DNA was used to perform the amplification of a conserved region of the 16S rDNA of the genus *Fusobacterium* (including *F. nucleatum*, *F. periodonticum*, *F. alocis* and *F. simiae*). The experiment was conducted in 20 μl reaction volumes using the KAPA HiFi Universal kit (KAPA Biosystems, USA). Thermocycling conditions were set as follows: 50 °C for 2 min, 95 °C (10 min) and 40 cycles of 15 s at 95 °C and 1 min at 60 °C. Amplification was performed on a ViiA 7 Real-Time PCR System (Applied Biosystems, Foster City, USA). All samples were tested in triplicates. For normalization, total DNA was measured using a Qubit 3.0 fluorometer combined with a dsDNA High-sensitivity Assay kit (Thermo Fisher Scientific, Waltham, MA, USA). QPCR C_t_ values were normalized by input material using the following formula:

$$ {\mathrm{Ct}}_{\mathrm{measured}}-{\log}_{\mathrm{efficiency}}\times \left(\mathrm{dilution}\ \mathrm{factor}\right)={\mathrm{Ct}}_{\mathrm{norm}} $$

C_t_ values were further converted into concentrations and subsequently into a theoretical copy numbers using the following formula:

$$ \left({\mathrm{Exp}}^{\left(\mathrm{Ctnorm}\hbox{-} \mathrm{b}\right)/\mathrm{a}\Big)}\times \mathrm{NA}\right)/\left({\mathrm{Fusobacterium}}_{\mathrm{bp}}\times \left(1\times {10}^{{{}^{\hat{\mkern6mu}}}^9}\right)\times {\mathrm{MW}}_{\mathrm{bp}}\right) $$

All resulting values were compared using the Mann-Whitney U-test.

### Data processing and statistical analysis

Raw datasets were fed into the QIIME pipeline [29] with standard OTU (operational taxonomic unit) picking parameters in closed-reference mode. Alpha and beta diversities were computed using a rarefaction depth of 10,800 sequences. Resulting abundance tables were analysed using the LefSe pipeline [30]. Briefly, a first step compared the relative abundance of all identified taxa between population groups using the Kruskal-Wallis test. In case of statistical significance (*P* < 0.05), a pairwise Wilcoxon test was conducted to analyse whether the feature is evenly distributed among individuals of the same group but with different demographic characteristics (e.g. different age, sex). All variables passing both tests are ranked using a Linear Discriminant Analysis model by their relative differences among groups.

## Results

### Composition of the gut microbiota in pre-treatment samples at the phylum and family levels

**Fig. 1:**
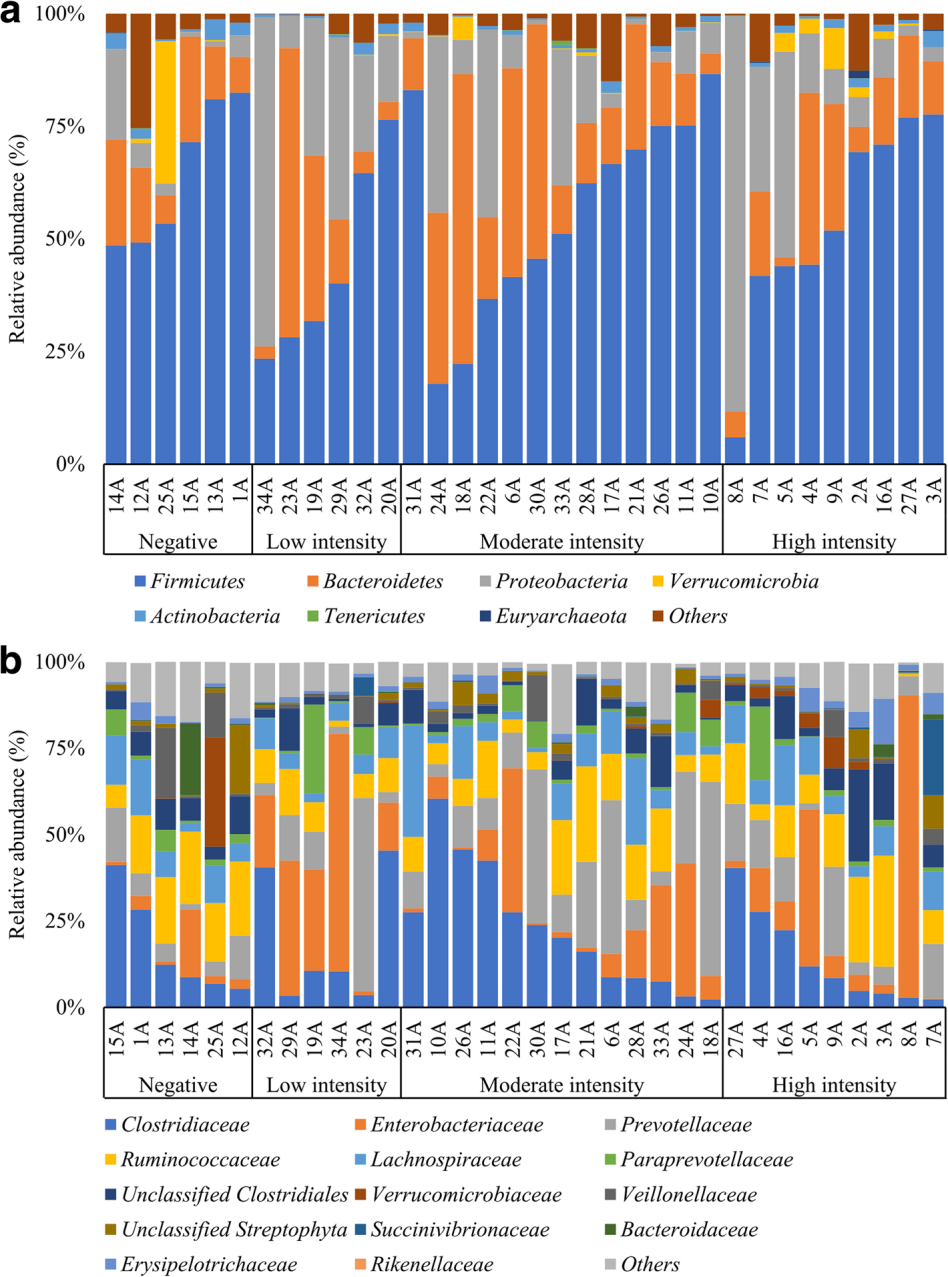
Composition of the microbiota of pre-treatment samples at two taxonomic levels. This bar chart shows the composition of the most abundant bacterial groups of each patient, both at the phylum level (**a**) as well as at the family level (**b**)

Pre-processing, including filtering and de-noising of the sequence datasets resulted in the analysis of a total of 4,160,032 sequences for 34 baseline samples with an average of 122,353 (Standard deviation: 74,507) sequences per sample. Results, presented at the phylum (Fig. 1a) and at the family (Fig. 1b) level of resolution are stratified according to infection intensities (negative, low, moderate and high).

Across all groups, the phyla *Firmicutes* and *Bacteroidetes* were the most abundant (> 50%), except in one sample from the low-intensity infection group (sample 34A) and two from the high-intensity infection group (samples 5A and 8A). The minimum and maximum observed abundance of *Firmicutes* were 5.9% and 86.5%, respectively. For the *Bacteroidetes*, the range spanned from 1.9% up to 64.2%. The ratio *Firmicutes* over *Bacteroidetes* remained above one across all but 6 samples (samples 6A, 18A, 19A, 23A, 24A and 30A). All 6 samples with ratios lower than one were found among the group of schistosome-infected individuals. The *Proteobacteria* phylum was the third most abundant taxon, with a median of 7.71% across all samples. While the relative abundance of this group was low in the control group (non-infected children), with a median of 3.69% (minimum of 1%; maximum of 20%), the median value in children infected with *S. mansoni* was 9.04% (minimum of 0.9%; maximum of 87%). The ratio of *Firmicutes* over *Proteobacteria* was above one in all except for 6 samples from the infected group (samples 5A, 8A, 22A, 24A, 29A and 34A). The ratio of *Bacteroidetes* over *Proteobacteria* was below one in the same samples, along with 6 additional samples from the *Schistosoma*-positive group (samples 7A, 10A, 20A, 28A, 32A and 33A). A final observation at the phylum level was the presence of *Actinobacteria* and *Verrucomicrobia* with a median of 0.9% and 0.1%, respectively. At the family level, the abundance of *Enterobacteriaceae*, a subgroup of the phylum *Proteobacteria* that contains numerous pathogens, reached a maximum of 20% in the control group (minimum of 2.6%) whereas in infected children, the abundance of the same family was as high as 87.6% of the overall composition. The abundance of members from the family *Prevotellaceae* was highly heterogeneous among the different groups, ranging from 1.6 to 56.2%.

### Comparison of the gut microbiota in *S. mansoni*-infected and non-infected children

**Fig. 2:**
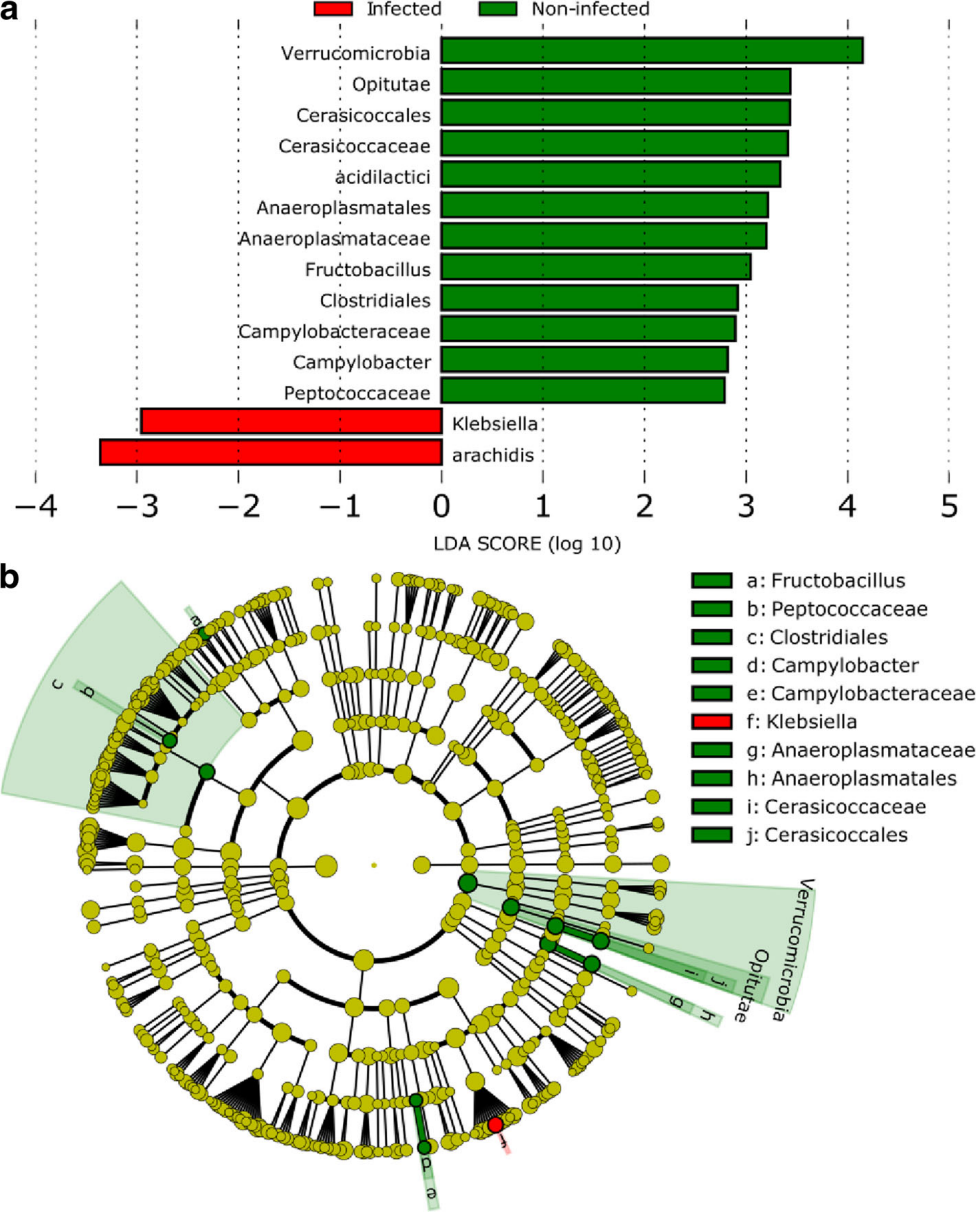
The gut microbiome composition differences in samples collected from *S. mansoni* negative and positive children at baseline. **a** Quantitative representation of the differences in the gut microbial composition of both groups using a bar chart representation and an LDA model (*P* < 0.05). **b** Taxonomic representation of significantly different relative abundances. Discriminative features (*P* < 0.05) are observed at various taxonomic levels

Differences between infected and non-infected individuals were explored using the LefSe pipeline. Figure 2 shows the abundances of the different taxonomic groups between infected and non-infected samples.

Differentiating abundances between these groups are largely observable among the *Proteobacteria.* Bacteria from the genus *Klebsiella* and *Enterobacter arachidis* were significantly more abundant in children infected with *S. mansoni* (*P* < 0.05). Over-abundance of members from the families *Cerasicoccaceae*, *Anaeroplasmataceae*, *Campylobacteraceae* and *Peptococcaceae*, and of the genus *Fructobacillus* seem to be significantly linked to the faecal microbiome of schistosome-negative children (*P* < 0.05).

### Comparison of the gut microbiota diversity among the different treatment groups

**Fig. 3:**
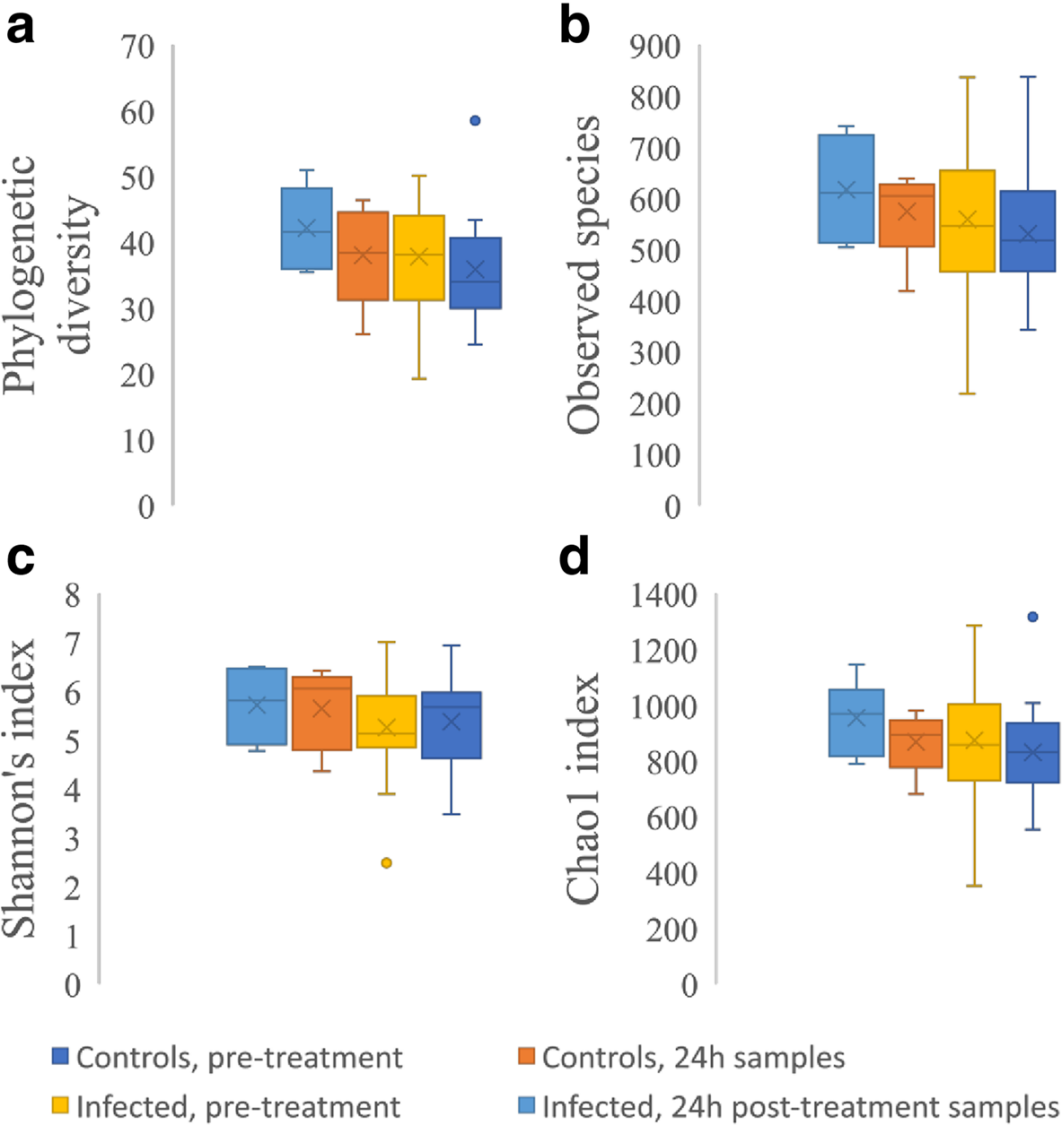
Indicators of microbial diversity in control and infected samples, both before and 24 hours after treatment. **a** Faith’s Phylogenetic Diversity indicator. **b** The absolute number of observed species. **c** The Shannon’s index. **d** The Chao 1 index

In Fig. 3, we present the different indicators commonly used to assess microbial diversity, obtained with the QIIME pipeline.

Although the diversity was lower in infected children, the range of diversity indicators was more heterogeneous in that group than in uninfected children. The variability in control samples at both sampling times (0 h and 24 h) ranged between 25.9–50.9, 419.1–742.1, 4.4–6.51 and 681.3–1147.8 for phylogenetic diversity, observed species, Shannon’s index and Chao 1 index, respectively. Identical indicators range between 19.2–50.1, 217.8–837.8, 4.5–7.0 and 352.6–1285 in samples from infected children, both at baseline and 24 h post-treatment. We did not observe any age-related differences (pre-school *vs* school-aged children).

### Association between praziquantel treatment and the gut microbiota composition

**Fig. 4:**
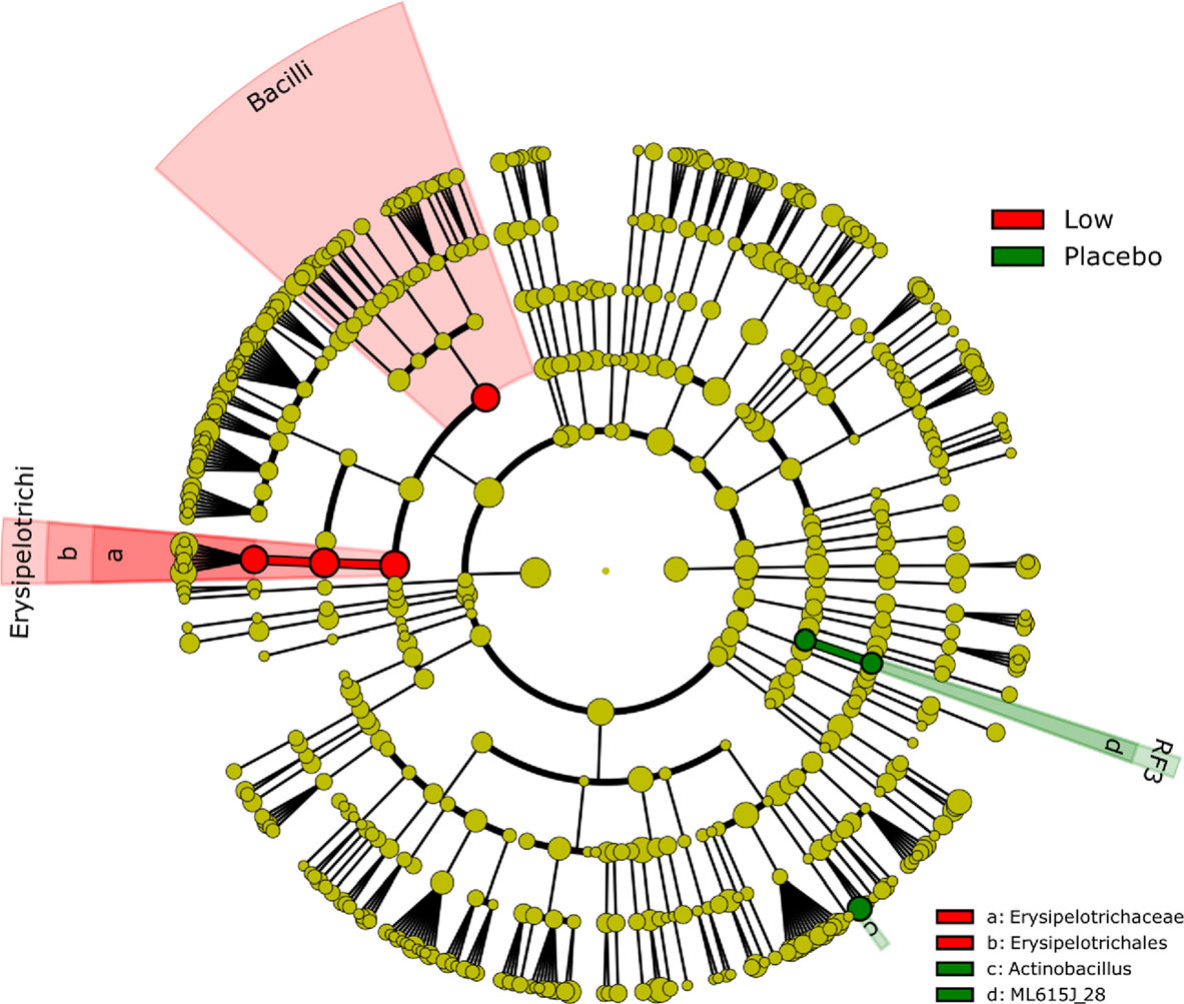
Differences in the gut microbiota of children infected with *S. mansoni* after administration of placebo or praziquantel. This cladogram shows the differences in bacterial composition of infected children receiving a placebo treatment (= Placebo) or infected children with failed treatment receiving a unique dose of 60 mg/kg praziquantel (= Low)

For the 34 samples collected 24 hours after treatment, sequencing resulted in the analysis of a total of 4,595,622 reads, with an average of 135,165 curated reads (Standard deviation: 81,880) per sample. To elucidate the potential off-target effects of praziquantel on the microbiota composition, 24-hour samples from those who received praziquantel (non-cured) *versus* those that received placebo were analysed. The taxonomic representation of the differential abundances is presented in Fig. 4.

Comparison of both treatment groups highlighted a few differences including a higher abundance of *Bacilli* and *Erysipelotrichi* taxa in volunteers who received praziquantel while some bacteria from the ML615J_28 order and the genus *Actinobacillus* were more abundant in the placebo group.

### Comparison of the gut microbiota of individuals with different praziquantel treatment outcomes

**Fig. 5:**
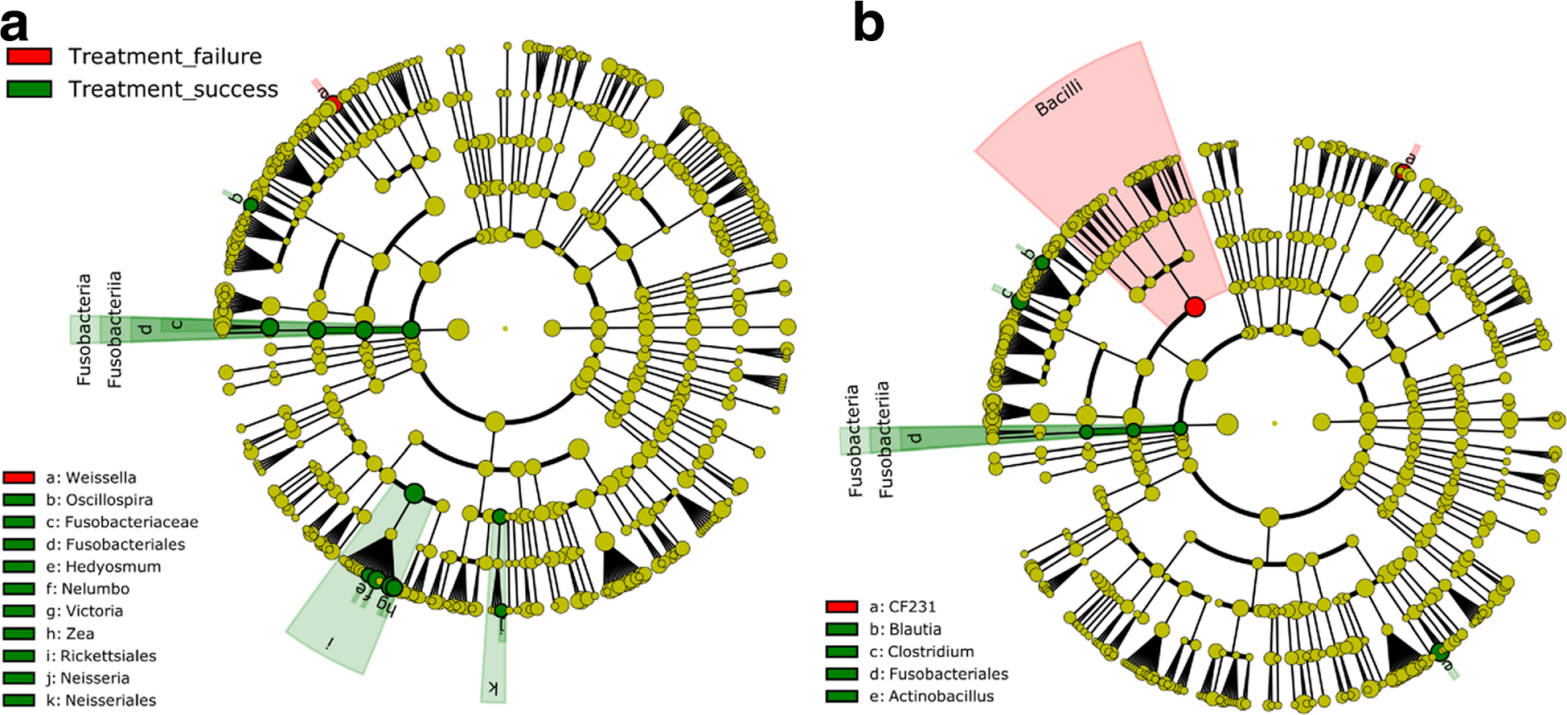
Comparison of microbiome in the treatment failures *versus* successful treatment groups. **a** Before administration of praziquantel. **b** 24 hours after administration of 60 mg/kg praziquantel. Using the same statistical tests did not reveal any differences of the same groups for the 3 weeks follow-up samples (data not shown)

Infected children were categorized after administration of a single dose of praziquantel in two groups, namely, (i) cured and (ii) not cured. Figure 5 summarizes the main differences between both groups.

**Fig. 6:**
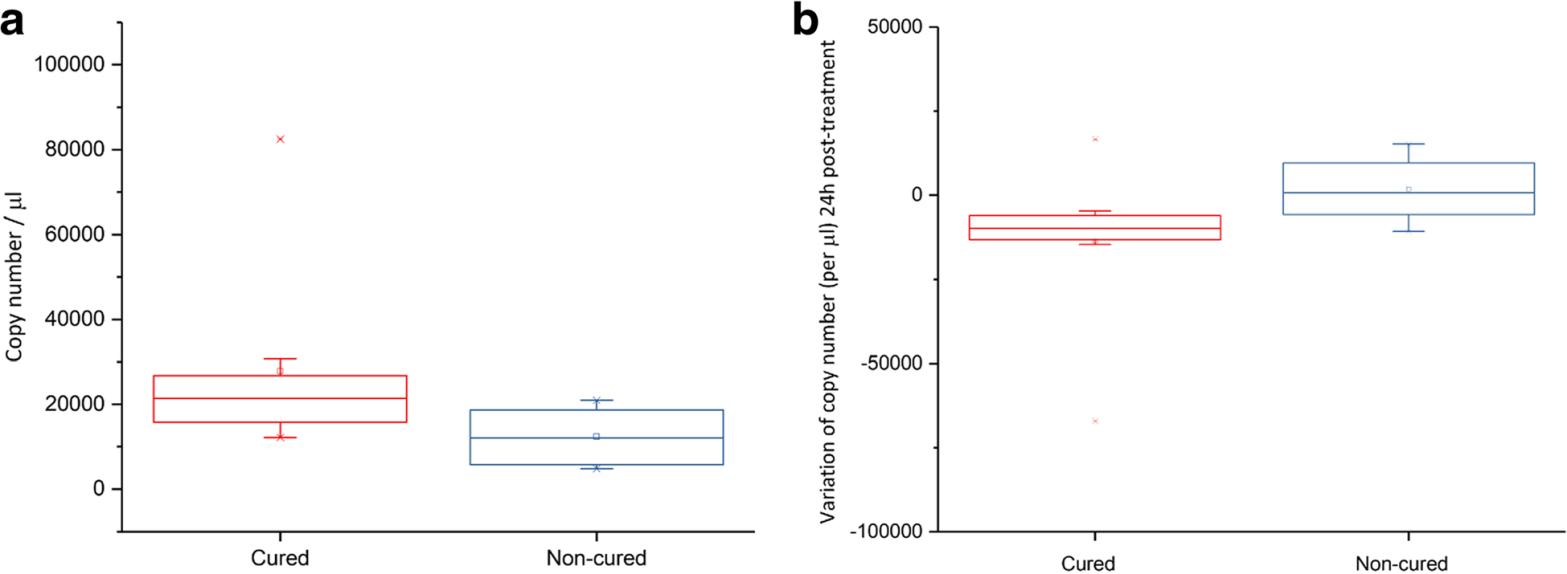
*Fusobacterium* spp. qPCR. **a** The copy number for *Fusobacterium* spp. before treatment both in cured and non-cured children. **b** The variation in copies number over the 24 hours post-treatment period in both cured and non-cured children

For samples collected at baseline, before treatment, there was an overabundance of members from the classes *Fusobacteriales*, *Rickettsiales* and *Neisseriales* in volunteers with high treatment efficacy. In contrast, individuals that were not cured, harbored in their microbiome a higher abundance of bacteria belonging to the genus *Weissella*. There was a significantly higher abundance of members from the class *Fusobacteriales* as well as of members from the genera *Clostridium* and *Actinobacillus* in cured volunteers, 24 hours after administration of praziquantel. At this time point, in children characterized by treatment failure, there was an overabundance of members from the class *Bacilli*. We used qPCR to confirm the results observed by *16S* PCR for the family *Fusobacteriaceae*, by selecting an assay to detect *Fusobacterium* spp., the most-represented genus from this family in our dataset (Fig. 6).

The measured qPCR efficiency, on the DNA of *Fusobacterium* *nucleatum* was 91.5%. Fluorimeter quantification of qPCR input is shown in Additional file 1: Table S1. *Fusobacterium* spp. is significantly more abundant in cured children before treatment (*P* = 0.018). The median normalized cycle threshold (C_t_) increase was +2.42 cycles between pre- and 24 hours post-treatment in fully cured children, equivalent to a median decrease in copy number is of -11,091 copies. In the population of children for which the treatment failed, the C_t_ value remained stable over the 24 h period, with a median of -0.17 cycles. The median C_t_ was 29.37 and 29.72 in the cured and non-cured groups, respectively, in the 24 hours post-treatment samples.

We compared the C_t_ values in placebo samples, to see whether associations exist between egg reduction rate and *Fusobacterium* spp. abundance. In the samples that showed an increase in *Fusobacterium* spp. abundance over the 24 hours (*n* = 5 samples; median = +1.21 cycle), the percentage of remaining eggs at follow-up was 66% while in those who had a decrease in *Fusobacterium* spp. abundance (*n* = 6 samples; median = -3.02 cycle), the average percentage of remaining eggs in the follow-up samples was 34%.

## Discussion

In this study, we describe for the first time the gut microbiome changes in *S. mansoni*-infected children and their potential impact on treatment outcome based on oral praziquantel application in an endemic rural area in Côte d’Ivoire.

### Associations between schistosomiasis and the gut microbiome

Four bacterial phyla are considered to dominate in the human gut microbiome, including *Firmicutes*, *Bacteroidetes*, *Actinobacteria* and *Proteobacteria* [12, 31]. The comparison of the gut microbiota profiles of children from the health district of Azaguié in Côte d’Ivoire, whether they were infected with *S. mansoni* or not, confirmed this finding. Abundance ratios of these four dominating phyla are usually good indicators for dysbiosis and have been linked to various health conditions, including colitis and metabolic disorders [32, 33]. Relative abundances of the different bacterial phyla depicted in this study correspond to those expected in normal human gut structure, with *Firmicutes* > *Bacteroidetes* > *Proteobacteria*, except for six children infected with schistosomes. While *Proteobacteria* are commensals of the gut microbiome, they are usually present in lower abundance than *Firmicutes* and *Bacteroidetes* in healthy individuals [32]. An outgrowth of bacteria from this taxon is typical of a major dysbiosis with clinical relevance, as several bacterial species from this phylum have been linked with pathogenesis in humans, resulting in either severe disease burden and/or death [32, 34–36]. In this specific population, the cumulated relative abundance of *Firmicutes* and *Bacteroidetes* represents more than 50% of the total bacterial composition in all but three samples. Interestingly, all three children, characterized by an untypical microbiome present a clear dysbiosis towards the phylum *Proteobacteria* and in all cases, the clinical assessment showed an outcome of clinical relevance, be it blood in stool and splenomegaly, simple splenomegaly or vomiting within three hours after praziquantel administration (data not shown). At the family level, we observed that members from the clade *Prevotellacae*, another important group for healthy gut functionality and which has been linked to various chronic diseases (e.g. rheumatoid arthritis or obesity), has a highly heterogeneous distribution among our samples [37, 38].

Diversity indices commonly used in ecological research, including the phylogenetic diversity, the number of observed species, the Shannon’s and the Chao 1 indices, are accepted indicators for dysbiosis [39–42]. In our study, we observed a trend that these different indicators show an overall lower bacterial diversity in children infected with *S. mansoni*. However, the inter-individual diversity varies more in this group and could reflect a less stable gut microbiota in infected children. This result fills a gap in our knowledge of associations of intestinal parasites and the gut microbiota structure. To date it has been shown that different intestinal parasites might increase, decrease or have no effect on the gut microbial diversity [6, 7, 43].

Associations between *S. mansoni* infection and the usual indicators of a healthy gut microbiome are relatively subtle. While we observe modifications that seem to be correlated with the presence of schistosomes, both in the phyla ratios and diversity indicators, the same modifications are not distributed homogeneously among all infected patients. This suggests that the gut microbiota stability is affected to some extent by the infection with *S. mansoni*, but that other factors, including the host’s specific genetic makeup in combination with environmental factors, such as nutrition, play a greater role in the composition of the gut microbiome, in this context. This observation is in partial agreement with the conclusion of the recent study of Kay et al. [44], stating that infection with *Schistosoma haematobium*, although being a bladder infecting schistosome, is associated with variations in the gut microbiota.

### Effect of praziquantel administration on the gut microbiome

Adverse effects from the orally administered praziquantel are usually mild or moderate and there are gut-related side effects of praziquantel (abdominal pain, cramps, with or without nausea and vomiting) that could potentially be related to a drug-induced microbial dysbiosis [45]. We compared the gut microbiotas of children 24 hours after administration of either a placebo or a high single praziquantel dose (60 mg/kg). The clades *Erysipelotrichi* and *Bacilli* were overabundant in stools produced by praziquantel-treated children. For the clade *Erysipelotrichi*, this information correlates with already published data, which states that this clade might be overabundant in patients receiving an antibiotic treatment [46]. Some members from the class *Bacilli* were shown to be potentially highly efficient xenobiotic metabolizers [47, 48]. However, since we did not study these bacteria at a lower taxonomic level, we cannot draw a more precise conclusion about this specific effect. At the time of this study, no published data were available linking either *Erysipelotrichi* or *Bacilli* clades to gastrointestinal complications. Therefore, since the structural changes in microbiota composition remain moderate and restricted to these two clades, we hypothesize that they do not contribute to the gut-related side effects of praziquantel administration. We can therefore conclude that praziquantel-related side effects are not related to indirect effects on the gut microbiome and that they are mostly explained by other factors, potentially including expulsion of dead *S. mansoni* adult worms, host’s genetics and/or degradation of *S. mansoni* eggs triggering the host’s inflammatory response, as previously shown [49, 50]. This observation, while based on a restricted amount of samples, gives new insights on conclusions made in a previous study on off-target effects of praziquantel [44]. While the study of Kay et al. [44] shows that praziquantel does not have an effect on the gut microbiota in the long term (12 weeks post-administration), we complement this information by stating that praziquantel does in fact have a slight short-term effect (24 hours post-administration) on the gut microbiota.

### Differences in the microbiome of successful *versus* failed treatments

As a final part of this study, we investigated potential bacterial factors in the gut microbiome that might explain the variations in treatment response to praziquantel. For this purpose, we stratified the samples from infected children into two groups based on the cure rates observed within the 3-week follow-up period. Of note, non-cured children showed on average an egg reduction rate of < 60%, which is way below the reference drug efficacy value of ≥ 90% set by WHO, hence praziquantel did not fulfil the criteria of clinical efficacy in these children. However, key pharmacokinetic parameters (AUC, Cmax) did not show a significant difference between cured and not cured children (data not shown) hence we can rule out that children did not take the treatment.

At baseline, the main differences were driven partly by endosymbionts of plant cells from the classes *Rickettsiales* and *Neisseriales*. For both classes, there is no specific literature describing their potential to modulate xenobiotics metabolism. Members from the class *Fusobacteriales* were also significantly more abundant in the successfully treated group at baseline. Twenty-four hours post-treatment, it is interesting to note that members from the same class were still significantly more abundant in the treatment success group. The qPCR assays confirmed a significant difference in the abundance of the genus *Fusobacterium*, a genus populated with potential pathogens, in the pre-treatment samples, between cured and non-cured patients [51]. This genus is particularly interesting as it includes species known to have inflammatory properties and species that have been linked to chronic disease, such as *Fusobacterium* *nucleatum*, that could be closely associated with the worm-triggered inflammatory response [52, 53]. Our results indicate that high *Fusobacterium* spp. abundance before treatment and high decrease rate of the bacteria over the first 24 hours post-treatment is correlated with a better outcome of praziquantel treatment. This could indicate that *Fusobacterium* spp. abundance and *S. mansoni* infection are dynamically linked and that preliminary presence of *Fusobacterium* spp. might condition the outcome of schistosomiasis treatment while being independent from the drug itself.

### Limitations

While this study does show preliminary results on gut composition related differences that may explain discrepancies in treatment outcome with praziquantel, it remains based on this specific paediatric population and a larger cohort is needed to confirm these results. For instance, additional patients with low-intensity of infection and high cure rate would be needed to draw significant conclusions as to what pertains the impact of different intensity of infections with *S. mansoni* on the gut microbiota. Also, additional investigations are required to fully understand the potential role and mechanism of action of these factors on praziquantel efficacy against schistosomiasis.

## Conclusions

This study highlighted three aspects of the interrelations between *S. mansoni*, praziquantel and the gut microbiota, namely; (i) the associations of *S. mansoni* and the usual indicators for a healthy gut microbiome seem to lay more in subtle modifications than in major compositional shifts. While we observed some modifications in the gut microbiome that seem to be specific to the presence of *S. mansoni*, the same modifications were not distributed evenly among infected patients; (ii) praziquantel administration had, similarly, relatively moderate effects on the gut microbiome. The observed effects on the microbiota are limited to two bacterial groups for which there is, so far, no proof of pathogenesis in human in the literature, but rather associations with responses to drug treatment. Therefore, we conclude that the gastrointestinal side effects observed in praziquantel administration are not related to an off-target effect of the drug on the microbial communities in the gut; and (iii) we were also able to highlight a bacterial taxon that could potentially play a role in the variations observed for schistosomiasis treatment outcomes with praziquantel, namely *Fusobacterium* spp. Further investigations are needed to understand and characterize the role of *Fusobacterium* spp. and their role in oral praziquantel treatment success.

## Additional files

Additional file 1: **Table S1.** Additional information about the children included in this study. (XLSX 12 kb)

Additional file 2: **Table S2.** Barcodes and DNA concentrations. Summary table showing the pooling barcodes and pre-sequencing DNA concentrations. (XLSX 10 kb)
